# Downregulation of peripheral *PTGS2*/COX-2 in response to valproate treatment in patients with epilepsy

**DOI:** 10.1038/s41598-020-59259-x

**Published:** 2020-02-13

**Authors:** Chitra Rawat, Rintu Kutum, Samiksha Kukal, Ankit Srivastava, Ujjwal Ranjan Dahiya, Suman Kushwaha, Sangeeta Sharma, Debasis Dash, Luciano Saso, Achal K. Srivastava, Ritushree Kukreti

**Affiliations:** 1 Institute of Genomics and Integrative Biology (IGIB), Council of Scientific and Industrial Research (CSIR), Mall Road, Delhi, 110007 India; 2 Academy of Scientific and Innovative Research (AcSIR), Council of Scientific and Industrial Research (CSIR), Mall Road, Delhi, 110007 India; 3 Department of Pharmacology, Faculty of Pharmacy, Jamia Hamdard, Delhi, 110062 India; 4 Institute of Human Behavior & Allied Sciences (IHBAS), Dilshad Garden, Delhi, 110095 India; 5 Department of Physiology and Pharmacology “Vittorio Erspamer”, Sapienza University, Rome, Italy; 6 Department of Neurology, All India Institute of Medical Sciences, Ansari Nagar, Delhi, 110029 India

**Keywords:** Transcriptomics, Neurological disorders

## Abstract

Antiepileptic drug therapy has significant inter-patient variability in response towards it. The current study aims to understand this variability at the molecular level using microarray-based analysis of peripheral blood gene expression profiles of patients receiving valproate (VA) monotherapy. Only 10 unique genes were found to be differentially expressed in VA responders (n = 15) and 6 genes in the non-responders (n = 8) (fold-change >2, p < 0.05). *PTGS2* which encodes cyclooxygenase-2, COX-2, showed downregulation in the responders compared to the non-responders. *PTGS2/*COX-2 mRNA profiles in the two groups corresponded to their plasma profiles of the COX-2 product, prostaglandin E_2_ (PGE_2_). Since COX-2 is believed to regulate P-glycoprotein (P-gp), a multidrug efflux transporter over-expressed at the blood-brain barrier (BBB) in drug-resistant epilepsy, the pathway connecting COX-2 and P-gp was further explored *in vitro*. Investigation of the effect of VA upon the brain endothelial cells (hCMEC/D3) in hyperexcitatory conditions confirmed suppression of COX-2-dependent P-gp upregulation by VA. Our findings suggest that COX-2 downregulation by VA may suppress seizure-mediated P-gp upregulation at the BBB leading to enhanced drug delivery to the brain in the responders. Our work provides insight into the association of peripheral *PTGS2*/COX-2 expression with VA efficacy and the role of COX-2 as a potential therapeutic target for developing efficacious antiepileptic treatment.

## Introduction

Epilepsy, a multifactorial neurological disease, affects about 69 million people worldwide constituting nearly 1% of the world population^1^. The treatment is primarily based on symptomatic pharmacological interventions i.e. antiepileptic drug (AED) therapy which controls the frequency of seizures in the patients. Despite the availability of appropriate therapy, there is significant inter-individual variability in AED response. Nearly 40–50% of the patients with epilepsy (PWE) fail to respond to their first AED monotherapy^2,3^ with 30% cases showing refractoriness^4^. This non-responsiveness to AEDs encouraged researchers to identify the predictors of poor response in PWE.

Clinical factors such as early age at onset, higher pretreatment seizure frequency, cryptogenic epilepsy, brain neuroanatomic abnormality, etc. have previously been observed to be potential predictors of poor response to prescribed AEDs^3,5^. Several studies also investigated the pharmacogenetics of AEDs and suggested the involvement of various drug-metabolizing enzymes (CYPs), drug transporters (ABC transporters), and drug target genes (ion-channels in brain) in deciding treatment outcome, however, remained inconclusive due to the inconsistency in their findings and failure of replication in different populations^6^. One reason behind this failure could be the multifactorial nature of the disease which involves genome-environment interactions and therefore limits the independent use of genetics in studying AED response^6–8^. Since any change in genetic or environmental factors ultimately leads to alterations in the gene functions, studying large-scale gene expression profiles may be beneficial in identifying the marker/s of variable AED response. However, the inaccessibility and invasiveness of the brain tissue sampling is a major limitation in investigating the changes at the target site i.e. brain. Peripheral blood, at present, is a tissue of choice for gene expression marker identification for various neurological as well as neuropsychiatric diseases^9–13^ as it shares several biological pathways with the brain. Some of the blood genomic expression studies also identified biologically relevant genes and pathways responsible for differential drug response^14–16^. However, the limited number of such reports and their low sample size leads to non-availability of substantial data to translate them clinically indicating the approach to be at a naïve stage and emphasizing the need for replication.

More than 24 AEDs are available in the market of which the conventional AEDs such as phenytoin (PHT), carbamazepine (CBZ) and valproate (VA) are the major prescriptions due to their cost-effectiveness^17^. Of these, VA is a first-line AED prescribed as monotherapy or in combination with other AEDs and is effective against a broad spectrum of seizure types including focal as well as generalized seizures^18^. The mechanism by which VA exerts its anticonvulsive effects is still unknown, but in epilepsy, it is assumed to increase gamma aminobutyric acid (GABA) concentrations in the brain^19^. However, to date, attempts to correlate increased GABA concentrations to the anticonvulsive activity of VA have remained challenging^20^. Alike other AEDs, VA is associated with significant inter-patient variability in seizure-free rates^21^. Identifying the molecular factors and biological pathways underlying this variability in VA efficacy may, therefore, benefit a larger patient population due to its broad-spectrum action.

The current study aims to identify the peripheral blood gene expression signatures of variable response to VA. Furthermore, the identified biological pathway associated with VA efficacy was explored using an appropriate *in vitro* model system, i.e., human cerebral microvascular endothelial cells, hCMEC/D3. Investigating gene expression signatures and underlying biological pathways associated with AED efficacy may provide valuable information to overcome pharmacoresistance in epilepsy.

## Results

**Figure 1:**
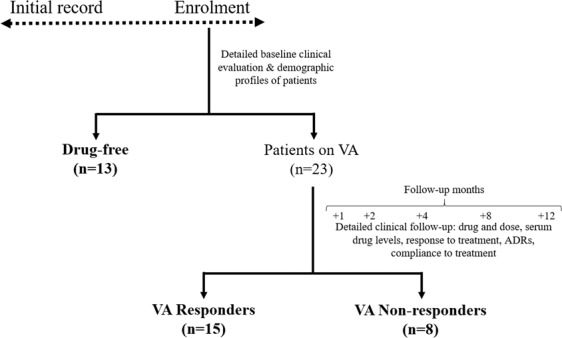
Patient selection and follow-up: A total of 36 patients with epilepsy were enrolled from the Outpatient Department of Neurology, IHBAS with detailed baseline clinical and demographic profiles. At the time of enrolment, 13 patients were “Drug-free”. The rest 23 patients were on VA therapy who were followed-up over a period of one year for drug, dose, serum drug levels, response to treatment, ADRs, compliance to treatment. During the course, patients who remained seizure-free were categorized as “VA Responders” (n = 15) and who experienced at least 3 seizures were categorized as “VA Non-responders” (n = 8). (n, number of cases; VA, valproate; ADRs, Adverse Drug Reactions).

The study consisted of 36 patients with idiopathic epilepsy: 13 “Drug-free”, 15 “VA Responders” and 8 “VA Non-responders” (Fig. 1). Blood samples were collected in predetermined daytime hours to avoid the effect of diurnal variation. Though a previous microarray-based study^21^ found no difference in the blood mRNA expression profiles obtained prior to and 24 h after a single seizure, few other reports have revealed altered production of acute phase proteins in plasma/serum within 24 hours^22^ and within 72 hours^23^ following seizure. Therefore, patients who experienced their last seizure at least 72 hours before the sample collection were recruited for the current study to prevent interference of recent seizure activity on gene expression. Dose and serum drug profiles of all the patients on VA therapy were recorded at follow-up period completion and observed to have no difference between the responders and non-responders (Table 1). However, the male:female ratio and age range varied among the three groups and therefore, were needed to be adjusted during the microarray data analysis.

**Table 1:** Demographic and clinical characteristics of enrolled PWE.

Demographic and clinical characteristics	Drug-free (n = 13)	On VA therapy	
		VA Responders (n = 15)	VA Non-responders (n = 8)
Male: Female ratio	7/6	9/6	4/4
**Age**, median (IQR), years	18.0 (14.0–23.0)	23.0 (19.5–26.0)	20.0 (17.0–28.0)
**Age at onset**, median (IQR), years	14.0 (13.0–18.0)	13.0 (12.0–16.0)	13.0 (7.0–17.0)
**BMI**, median (IQR), kg/m^2^	16.9 (15.8–19.4)	21.9 (19.1–25.4)	19.2 (18.0–22.1)
**Treatment duration**, median (IQR), months	—	22.0 (16.0–29.8)	20.5 (16.0–26.3)
**Maintenance dose [mg/day]**			
median (IQR)	—	800 (400–1000)	800 (800–1050)
**Dosage [(mg/day)/kg]**			
median (IQR)	—	16.3 (11.4–17.5)	16.7 (15.8–20.4)
**Serum drug level [mg/L]**			
median (IQR)	—	83.7 (76.1–103.4)	(72.2–96.2)
**Dose corrected serum drug level [(mg/L)/[(mg/day)/kg]]**			
median (IQR)	—	5.3 (4.9–10.1)	5.3 (4.6–6.5)

n: number of samples; IQR: Interquartile range; VA: valproate.

### *PTGS2*/COX-2, the most significantly downregulated gene in “VA Responders”

The background-corrected expression data was filtered for probes with a detection p < 0.05 for at least 50% arrays per chip which reduced the initial 47,231 probes to 13,165 probes that were eventually considered expressed. The expressed probes were then log_2_ transformed and normalized using the Supervised Normalization of Microarrays (SNM) method^24^ where the effect of the biological variable of relevance, i.e. drug response, was retained and effects of the other probable confounding factors such as sex, age, and other technical variables like batch effects, intensity-dependent effects, etc. were adjusted. To determine the differences in the effect of VA in the responder and non-responder groups, the profiles of these groups were compared with that of the drug-free patients, instead of comparing with each other. Therefore, the differential gene expression analysis was performed with two comparison groups: (i) VA Responders vs Drug-free, and (ii) VA Non-responders vs Drug-free. At fold change, FC > 2 and p < 0.05, the analyses resulted in 14 differentially expressed genes (DEGs) in “VA Responders” and 10 in “VA Non-responders” among which 4 genes were common between the two groups (Supplementary Table 1) and therefore were discarded from further analysis. Among the remaining unique DEGs, only one gene, *PTGS2* (encodes cyclooxygenase-2, COX-2) in “VA Responders” and one gene, *GNLY* in “VA Non-responders” were found to remain statistically significant even after the false discovery rate, FDR < 0.10 adjustment (Table 2). Quantitative reverse transcription-polymerase chain reaction (qRT-PCR) successfully validated microarray expression profiles of all the unique DEGs (Table 2). A multiclass comparison of *PTGS2* expression in the three groups using receiver operating characteristic (ROC) analysis showed an area under the curve (AUC) of 0.663 indicating a moderate diagnostic performance. The AUCs for “Drug-free vs VA Responders” and “VA Responders vs VA Non-responders” comparisons were 0.764 and 0.733, respectively (Supplementary Fig. 1).

**Table 2:** Unique DEGs in “VA Responders” and “VA Non-responders” (FC > 2, P_uncorrected_ < 0.05) and correlation with qRT-PCR findings.

Probeset ID	Gene Symbol	Gene name	Cytoband	Microarray			qRT-PCR		Pearson’s correlation	
				P Value *(uncorrected)*	FDR	Fold Change	P value	Fold Change	r	P value
**VA Responders vs Drug-free**										
ILMN_2054297	*PTGS2/COX2*	Prostaglandin-endoperoxide synthase 2	1q31.1	0.0030	**0.0819**	−2.03	0.0197	−2.39	0.8235	<0.0001
ILMN_1705183	*MPO*	Myeloperoxidase	17q22	0.0079	0.1153	2.06	0.0188	2.14	0.7348	<0.0001
ILMN_2165289	*DEFA3*	Defensin, alpha 3, neutrophil-specific	8p23.1	0.0101	0.1259	3.18	0.0265*	3.45*	0.6382	0.0024
ILMN_2102721	*DEFA1B*	Defensin, alpha 1B	8p23.1	0.0114	0.1304	3.15				
ILMN_2193213	*DEFA1*	Defensin, alpha 1	8p23.1	0.0165	0.1516	2.64				
ILMN_1693262	*LOC653600*	Predicted: similar to Neutrophil defensin 1 precursor	8p23.1	0.0174	0.1541	3.45				
ILMN_1680424	*CTSG*	Cathepsin G	14q12	0.0265	0.1857	2.63	0.0229	3.12	0.7176	0.0007
ILMN_1712522	*CEACAM6*	Carcinoembryonic antigen-related cell adhesion molecule 6	19q13.2	0.0301	0.1919	2.15	0.0426	2.75	0.4993	0.0296
ILMN_1706635	*ELANE*	Elastase, neutrophil expressed	19p13.3	0.0342	0.2085	2.43	0.0343	2.17	0.5699	0.0087
ILMN_1753347	*DEFA4*	Defensin, alpha 4, corticostatin	8p23.1	0.0359	0.2133	2.56	0.0466	2.29	0.5140	0.0291
**VA Non-responders vs Drug-free**										
ILMN_1708779	*GNLY*	Granulysin	2p11.2	0.0002	**0.0726**	2.13	0.0211	1.92	0.6986	0.0009
ILMN_2399463	*VAV3*	Vav guanine nucleotide exchange factor 3	1p13.3	0.0030	0.1124	−3.49	0.0183	−4.28	0.8196	<0.0001
ILMN_1695404	*LY6E*	Lymphocyte antigen 6 complex, locus E	8q24.3	0.0054	0.1199	2.07	0.0220	2.63	0.3681	0.1330
ILMN_3309349	*SNHG8*	Small nucleolar RNA host gene 8 (non-protein coding)	4q26	0.0069	0.1267	2.06	0.0374	1.96	0.5047	0.0215
ILMN_1696584	*ORM1*	Orosomucoid 1	9q32	0.0140	0.1515	−2.31	0.0025	3.94	0.7432	<0.0001
ILMN_1781388	*PGM5*	Phosphoglucomutase 5	9q21.11	0.0211	0.1691	2.11	0.0211	1.92	0.7395	0.0025

^*^Variants of same gene; FDR: False discovery Rate; Bold signifies FDR < 0.10.

### Plasma PGE_2_ levels correlate with COX-2 mRNA profiles

COX-2 synthesizes prostaglandins, majorly PGE_2_, which can be quantified in plasma. In a recently published work from our group, we compared the plasma PGE_2_ levels in drug-free patients with different epilepsy subtypes as well as in responder and non-responder groups on different AED monotherapy^25^. The work involves the sample groups from the current study in which we observed lower levels in “VA Responders” compared to “Drug-free” (232.1 vs 489.3 pg/ml, p < 0.01) and to “VA Non-responders” (232.1 vs 611.9 pg/ml, p < 0.0001) indicating PGE_2_ levels to be associated with COX-2 mRNA expression profile. No significant difference was observed in “VA Non-responders” when compared with “Drug-free” group.

### Glutamate increases COX-2 activity without affecting its expression *in vitro*

**Figure 2:**
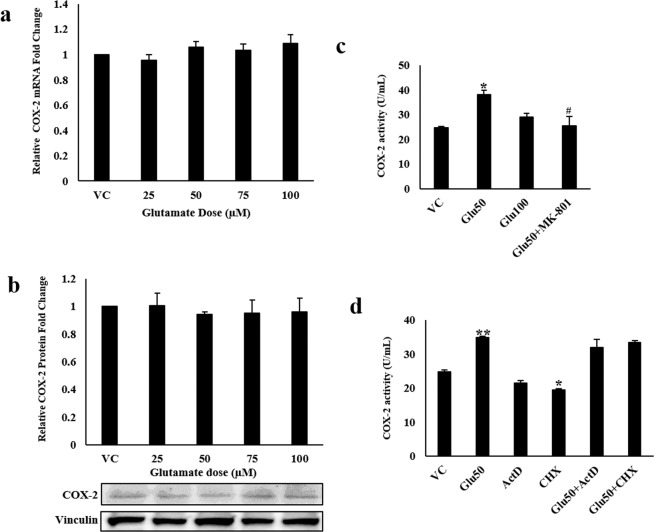
Effect of high concentrations of glutamate (Glu) on COX-2 expression and activity: hCMEC/D3 cells were treated with different concentrations of glutamate for 12 h. After treatment, cells were harvested to assess, **(a)** COX-2 mRNA levels by qRT-PCR, **(b)** COX-2 protein levels by immunoblotting, and **(c,d)** COX-2 activity. Cells in some of the wells were pretreated with MK-801 for 15 min, and with 10 µM ActD and 200 µM CHX, each for 45 min before treatment with 50 µM glutamate for further 12 h. Data were represented in mean ± S.D. of at least four independent experiments. (*p < 0.05, **p < 0.01, compared to VC; ^#^p < 0.05, ^##^p < 0.01, compared to glutamate-treated group).

Based on our microarray findings and the available literature, we hypothesized that VA may reduce P-glycoprotein (P-gp) activity by downregulating COX-2 at the blood-brain barrier (BBB), hence leading to its better delivery to the brain in the responders. However, due to the poor peripheral expression of P-gp-encoding gene, *ABCB1*, the effect of VA on the gene could not be studied in blood. Therefore, to establish our hypothesis, we selected the hCMEC/D3 cell line owing to its suitability for carrying out drug transport studies. To emulate seizure conditions, the cells were treated with high levels of glutamate (25–100 µM) in contrast to the normal levels i.e. 0.3–2 µM. No change was found in COX-2 mRNA as well as protein expression (Fig. 2a,b), however, COX-2 activity was observed to be increased by 50% (p < 0.05) after 12 hours of treatment with 50 µM glutamate (Fig. 2c). Pre-treatment with 10 µM of NMDA receptor antagonist, dizocilpine (MK-801) blocked the glutamate-mediated increase in COX-2 activity suggesting the involvement of NMDA receptor in the signaling cascade (Fig. 2c). When cells were treated with a known transcriptional inhibitor, 10 µM Actinomycin D (ActD), and a known translational inhibitor, 200 µM cycloheximide (CHX), 45 min. prior to glutamate treatment, both failed to prevent the increase in COX-2 activity by glutamate (Fig. 2d), substantiating the findings of the mRNA and protein expression data, thus dismissing the involvement of transcriptional or translational regulation of COX-2 by glutamate.

### Glutamate upregulates P-glycoprotein via COX-2/EP1 signaling

**Figure 3:**
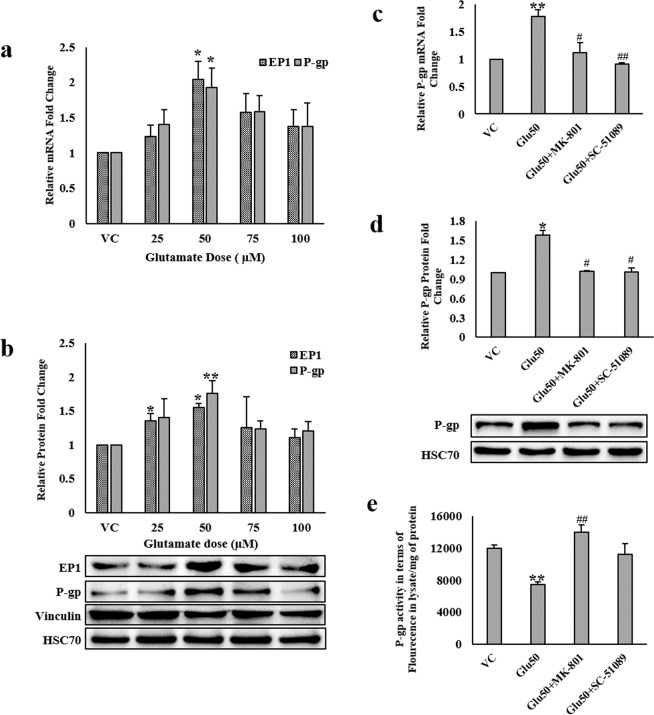
Effect of high concentrations of glutamate (Glu) on P-gp expression and activity: hCMEC/D3 cells were treated with different concentrations of glutamate for 12 h. After treatment, cells were harvested to assess, **(a,c)** EP1 and P-gp mRNA levels by qRT-PCR, **(b,d)** EP1 and P-gp protein levels by immunoblotting. Cells in some of the wells were pretreated with 10 µM MK-801 for 15 min, and with 40 µM SC-51089 for 20 min before treatment with 50 µM glutamate for further 12 h. **(e)** P-gp activity was measured on live cells after exposure to different chemicals for 12 h by rhodamine 123 efflux assay. Data were represented in mean ± S.D. of at least four independent experiments. (*p < 0.05, **p < 0.01, compared to VC; ^#^p < 0.05, ^##^p < 0.01, compared to glutamate-treated group).

To determine if glutamate-induced COX-2 activity regulates P-gp at the BBB via prostaglandin receptor EP1, we examined the effect of glutamate on EP1 as well as P-gp expression in hCMEC/D3 cells. Both EP1 and P-gp mRNA levels were increased by approx. 2-fold (p < 0.05) (Fig. 3a) while their protein levels were upregulated by 1.5-fold (p < 0.05) and 1.7-fold (p < 0.01), respectively (Fig. 3b). Exposure to NMDA receptor antagonist, MK-801 and EP1 receptor antagonist, 8-Chlorodibenz(Z)[b,f][1,4]oxazepine-10(11 H)-carboxylic acid 2-[1-oxo-3-(4-pyridinyl)propyl]hydrazide hydrochloride (SC-51089), prior to glutamate treatment prevented glutamate-induced increase in P-gp mRNA and protein expression (Fig. 3c,d) suggesting the involvement of NMDA and EP1 receptors in P-gp regulation. On measuring P-gp efflux activity, glutamate significantly increased rhodamine 123 efflux by 1.6 fold (p < 0.05) causing lower fluorescence inside the cells (Fig. 3e) which implies increase in P-gp function by glutamate. Both the antagonists, MK-801 and SC-51089 blocked glutamate-mediated efflux of rhodamine 123.

### Valproate inhibits glutamate-mediated P-glycoprotein upregulation by downregulating COX-2

**Figure 4:**
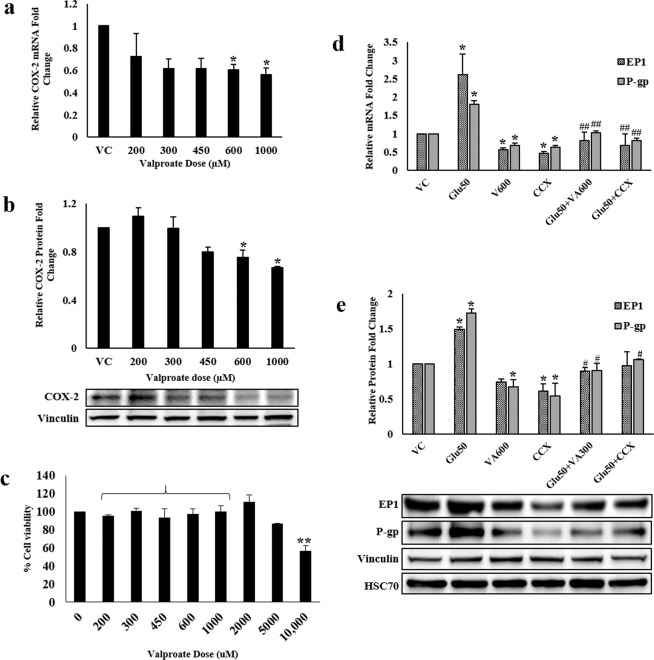
Effect of valproate (VA) on COX-2, EP1 and P-gp expression: Cells were treated with different concentrations of VA for 12 h. After treatment, cells were harvested to assess, **(a)** COX-2 mRNA levels by qRT-PCR, **(b)** COX-2 protein levels by immunoblotting. **(c)** Cell viability was measured upon treatment with different concentrations of VA for 24 h using MTT assay. **(d,e)** Cells in different wells were treated 50 µM glutamate, 600 µM VA, 2 µM CCX or their combination. In combinational treatments, cells were exposed to VA for 2 h and CCX for 30 min prior to treatment with 50 µM glutamate for further 12 h. After treatment, cells were harvested to assess EP1 and P-gp mRNA and protein levels. Data were represented in mean ± S.D. of at least four independent experiments. (*p < 0.05, **p < 0.01, compared to VC; ^#^p < 0.05, ^##^p < 0.01, compared to glutamate-treated group).

**Figure 5:**
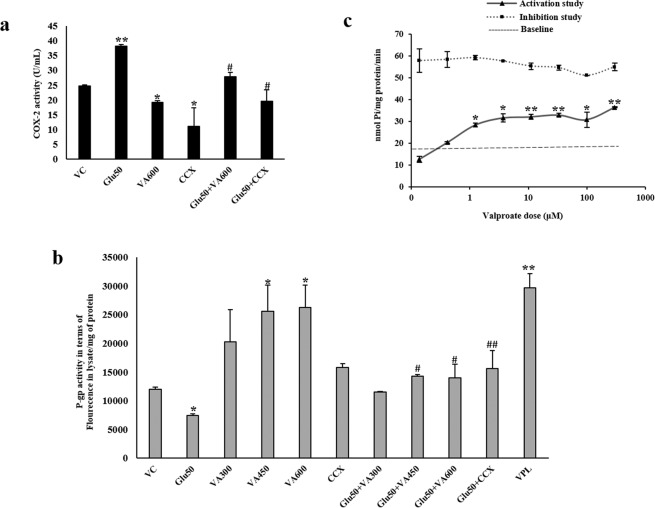
Effect of valproate (VA) on glutamate (Glu)-induced COX-2 and P-gp activity: (**a)** Cells in different wells were treated 50 µM glutamate, 600 µM VA, 2 µM CCX or their combination. In combinational treatments, cells were exposed to VA for 2 h and CCX for 30 min prior to treatment with 50 µM glutamate for further 12 h. After treatment, cells were harvested to assess COX-2 activity. **(b)** Cells in different wells were treated 50 µM glutamate, 300–600 µM VA, 2 µM CCX, 50 µM VPL or their combination. After treatment, P-gp activity was measured on live cells by rhodamine 123 efflux assay. **(c)** P-gp specific ATPase assay with increasing concentration of VA. Data were represented in mean ± S.D. of at least four independent experiments (three in case of Fig. 5c). (*p < 0.05, **p < 0.01, compared to VC/baseline; ^#^p < 0.05, ^##^p < 0.01, compared to glutamate-treated group).

VA was also evaluated for its effect on the regulation of COX-2 as well as P-gp in hCMEC/D3 cells. A concentration range equivalent to the therapeutic serum levels of VA in epilepsy patients i.e. 300–600 µM along with a lower concentration, 200 µM, and a higher concentration, 1000 µM, was taken into consideration for different experiments. VA treatment resulted in a concentration-dependent decrease in COX-2 expression (Fig. 4a,b). At the therapeutic concentration of 600 µM, VA reduced COX-2 mRNA amount by 1.65-fold and protein amount by 1.33-fold (p < 0.05). To determine whether VA’s downregulatory action on COX-2 was attributed to the cytotoxic effect of VA treatment, cell viability at different concentrations of VA was measured using 3-(4,5-dimethylthiazol-2-yl)-2,5-diphenyltetrazolium bromide (MTT) assay wherein no significant cell death was observed at the selected concentration range after 24 hours of treatment (Fig. 4c). Furthermore, VA and the well-known COX-2 inhibitor, celecoxib (CCX) significantly prevented glutamate-mediated EP1 and P-gp upregulation (Fig. 4d,e). Treatment with VA displayed 1.4-fold lower COX-2 activity in contrast to 2-fold decrease by CCX (Fig. 5a). Rhodamine 123 efflux assay also demonstrated diminished P-gp activity by VA as efficiently as by the known P-gp inhibitor, verapamil (VPL), further neutralizing the effect of glutamate (Fig. 5b). Taken together, our findings suggest that VA may negatively regulate P-gp via the COX-2/EP1 signaling cascade.

### ATPase assay shows valproate as a potential substrate of P-glycoprotein

The substrate interaction of VA with the transporter P-gp was performed using P-gp-specific ATPase assay where the liberation of inorganic phosphate, Pi was measured in the presence of VA. In the activation assay, increasing concentration of VA from 0.14–300 μM slightly stimulated ATPase activity from the baseline measurement (Fig. 5c) confirming a substrate relationship of VA with P-gp at the lower concentrations. The inhibition assay found no effect of VA on the liberation of Pi mediated by a known substrate demonstrating that VA does not inhibit P-gp ATPase activity at the selected concentration range. The assay could not be performed with a VA concentration beyond 300 μM due to the detection range limit of the assay.

## Discussion

Nearly 40–50% of the PWE receiving their first AED monotherapy display non-responsiveness, therefore, identifying the biomarkers of variable AED response is crucial. The current study identified two sets of blood gene expression signatures altered in (1) “VA Responders”, and (2) “VA Non-responders”, when compared to patients not receiving any treatment. We identified a set of 10 genes and 6 genes having unique blood gene expression patterns with FC > 2, p < 0.05 in “VA Responders” and “VA Non-responders”, respectively. Among these genes, *PTGS2*, which was found to be the most significant downregulated gene in “VA Responders”, remained significant after applying FDR correction of <0.1. Our result is consistent with an earlier report by Tang *et al*.^21^ which also reported downregulation of the same gene in a smaller subset of children with epilepsy responding to VA.

*PTGS2* encodes the enzyme COX-2 which synthesizes the proinflammatory mediators, prostaglandins and is involved in seizure-mediated neuroinflammation^26^. Several *in vivo* studies demonstrated COX-2 induction following a convulsive challenge^27–30^ and its pharmacological inhibition by COX-2 inhibitory drugs leading to enhanced efficacy of the prescribed AEDs and decreased seizure frequency^31–34^. Prostaglandins along with other inflammatory molecules are also often observed to be released by the astrocytes, microglia, brain endothelial cells, and peripheral immune cells affecting the function and neuroexcitability of the brain^35^. Since COX-2 majorly synthesizes PGE_2_ which is detectable in plasma, we measure the plasma PGE_2_ levels of the enrolled patients. “VA Responders” had significantly lower levels of plasma PGE_2_ compared to that of “Drug-free” and “VA Non-responders” which corresponded to their downregulated *PTGS2*/COX-2 mRNA levels obtained from gene expression profiling suggesting an association between COX-2 expression and VA response.

Mounting evidences suggest a key involvement of COX-2 in mediating BBB dysfunction, ultimately causing pharmacoresistance in epilepsy^34,36–38^. The dysfunctioning of the BBB is often associated with the induction of efflux transporters at the BBB which is a major obstacle to pharmacotherapy in various CNS diseases including epilepsy^39^. Elucidating the molecular mechanisms regulating these transporters is crucial to understand AED efficacy and to identify neurotherapeutic targets for developing better efficacious pharmacotherapy. P-gp, a multidrug efflux transporter, is highly expressed in brain capillary endothelial cells and its upregulation in the surgically-removed brain samples of PWE is widely acknowledged as the major cause for AED resistance^40,42–44^. It has been proposed that seizures induce COX-2 expression^45,46^ which further regulates P-gp by producing increased levels of PGE_2_^36,37^. PGE_2_ molecules bind to the EP1 receptor activating a signaling cascade leading to increased expression of P-gp at the BBB, thereby causing increased efflux of the prescribed AED/s and lower drug delivery to the brain resulting in poor efficacy^47^. *In vitro* investigations exploring the effect of different AEDs on P-gp expression and function in rat brain endothelial cells reported contradictory findings. While Yang *et al*.^48^ found an increased P-gp expression and function after long-term exposure to AEDs such as phenobarbital, PHT, CBZ and VA, study by Ambroziak *et al*.^49^ observed no effect in these cells, demanding the need to further explore the consequence of AED treatment in human brain endothelial cells. To our knowledge, the current study is the first to demonstrate the effect of VA treatment on P-gp expression and function in human brain endothelial cells. In our study, we observed significant downregulation of COX-2 mRNA levels in “VA Responders” which corresponded to their lower plasma PGE_2_ levels. In addition, our previous study observed no difference in the COX-2 mRNA levels between the drug-free patients with idiopathic epilepsy and healthy controls at FC > 2, p < 0.05^50^, suggesting that the downregulation in COX-2 expression profiles of the responders in the current study may be due to VA treatment. If COX-2 regulates P-gp, a downregulation in COX-2 might result in lower expression of P-gp as well. Based on these findings, we hypothesized that VA suppresses seizure-mediated P-gp induction by downregulating COX-2 at the BBB, thereby displaying enhanced efficacy in the responders.

To validate our hypothesis, we explored the biological pathway involved in COX-2-dependent P-gp regulation in a BBB *in vitro* model system, hCMEC/D3. In the study, we first demonstrated the effect of high concentrations of glutamate on the regulation of three relevant genes, (1) the gene of interest, COX-2, (2) the PGE_2_ receptor, EP1, and (3) the multidrug efflux transporter, P-gp. COX-2 activity was significantly increased by glutamate within a limited concentration range while its expression remained unaltered at both mRNA and protein level. Pre-treatment with the transcriptional as well as translational inhibitors showed no reduction in glutamate-induced COX-2 activity confirming the effect of glutamate on COX-2 to be independent of any transcriptional and translational changes. Contrary to our findings, previous preclinical^34,37,38^ and clinical^51–54^ studies have reported overexpression of COX-2 in brain and brain endothelial cells following seizures or in epilepsy. Epileptogenesis is a complex, multifactorial mechanism involving alterations in inhibitory and excitatory neurotransmitters, ion channels, neuroinflammatory pathways, etc., therefore, the effect on a particular gene may differ with the type of convulsive challenge to *in vitro* and *in vivo* model systems. Since our study investigated the pathway relevant to the brain endothelial cells which majorly activates the NMDA receptor pathway during a seizure event, we examined the effect of high concentrations of glutamate in the cells. A study by Zibell *et al*.^38^ also found no change in brain endothelial COX-2 expression of lithium-pilocarpine status epilepticus rat model and in isolated rat brain capillaries exposed to 50 µM and 100 µM glutamate and reported that seizure induction has no effect on COX-2 expression but causes enhanced substrate (arachidonic acid) flux to COX-2. However, our findings suggest that glutamate, via some unidentified mechanism, is causing an increase in COX-2 activity thereby generating increased amounts of PGE_2_.

The prostaglandin, PGE_2_ signals via four G protein-coupled receptors, EP1, EP2, EP3, and EP4, however, it binds principally to EP1 eventually causing P-gp upregulation. Our study observed increased mRNA and protein expression of both EP1 and P-gp in response to glutamate treatment. Rhodamine 123 efflux assay, a P-gp-specific transport assay, also showed induced P-gp activity in the hCMEC/D3 cells upon glutamate exposure which was significantly blocked by the NMDA receptor antagonist, MK-801 and EP1 receptor antagonist, SC-51089 confirming the involvement of the two receptors in glutamate-mediated transcriptional activation of P-gp. Several investigations demonstrated P-gp upregulation in brain and BBB in patients with refractory epilepsy^40–44^. Drug-resistant rodent epilepsy models were also reported to have higher brain P-gp levels than the drug-responsive ones^55^. Earlier attempts to elucidate the molecular mechanism of this seizure-induced P-gp upregulation discovered activation of glutamate-mediated endothelial NMDA receptor in rodent brain^36,38,56^ which was prevented by the administration of selective and non-selective COX-2 inhibitors^36–38^ suggesting the role of COX-2 in P-gp regulation. Such reports indicated that COX-2 activation during seizures may upregulate P-gp, thereby causing pharmacoresistance due to enhanced drug efflux out of the brain. Inhibiting COX-2 may, therefore, help in overcoming pharmacoresistance.

On the other hand, the anticonvulsant, VA caused a concentration-dependent decrease in COX-2 expression. It also prevented the glutamate-mediated EP1 and P-gp upregulation as efficaciously as the known COX-2 inhibitor, CCX. Functional assays showed diminished COX-2 as well as P-gp activity upon treatment with VA or CCX suggesting that VA may cause downregulation of P-gp via the COX-2/EP1 signaling cascade. Downregulation of COX-2 by VA has often been attributed to reduced binding of the transcription factor, NF-κB to the promoter region of COX-2. Rao *et al*.^57^ reported a decreased NF-κB DNA binding activity due to reduced protein levels of its p50 subunit upon chronic treatment of VA in rats while Chuang *et al*.^58^ suggested that VA activates MKP-1 to dephosphorylate p38MAPK and JNK, leading to decreased NF-κB-p65 and C/EBPβ binding to the COX-2 promoter in lipopolysaccharide-stimulated murine brain endothelial cells. Also, VA is a known histone deacetylase (HDAC) inhibitor, a property reported to be responsible for its neuroprotective and anti-inflammatory activities^58–60^. HDAC inhibitors have previously been observed to suppress COX-2 via their action on associated transcription factors^58,61^ and therefore, need to be explored for potential anticonvulsive effect. The ATPase assay in the current study also showed a drug-transporter interaction between VA and P-gp indicating VA to be a substrate of P-gp. P-gp shows a broad substrate specificity with most of the substrates being organic and hydrophobic in chemical structure and are positively charged at neutral pH^62^. In addition, aromaticity and neutral or weakly basic nature are other common structural denominators for P-gp substrates. However, some weak acids such as methotrexate, PHT, etc. can also be transported, albeit at a slow rate^63^. VA, an organic weak acid, has previously been shown not to be a substrate for P-gp^64^ which contradicts the findings of the ATPase assay of the current study. The conflict in the findings may be due to the difference in the selected concentration range of the experiments in the two studies. Our study presented the data at the VA concentration below 300 μM due to the detection limit of the assay while the study by Baltes *et al*.^64^ demonstrated their experiments at 280–690 μM concentration. VA has also been observed to have a P-gp inhibitory potential at a dose of 250 and 500 μM^65^. Earlier, Polli *et al*.^66^ reported that compounds that inhibit Pgp at higher concentrations may become substrates at lower concentrations. If this is the case, our findings do not provide sufficient proof that VA is a substrate for P-gp. Therefore, the findings of the present study need to be validated using confirmatory assays measuring the intracellular accumulation of test drugs at a broader concentration range.

**Figure 6:**
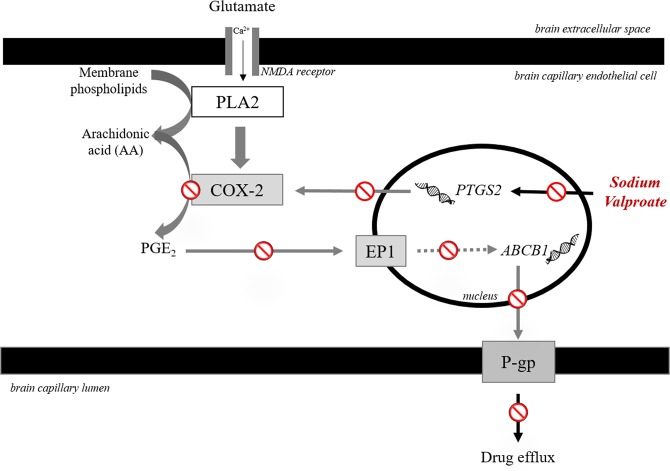
Proposed mechanism of valproate (VA) response in epilepsy: During seizure, high levels of glutamate binds to the endothelial NMDA receptor causing Ca2+ influx via the receptor activating phospholipase A2 (PLA2) which generates arachidonic acid (AA) from the membrane phospholipids. COX-2 acts as a key downstream effector producing high amounts of the inflammatory mediator, prostaglandin E2 (PGE_2_) from AA. PGE2 then binds to its key receptor, EP1 activating an unidentified cascade of signaling events driving the transcriptional activation of P-glycoprotein (P-gp), thereby leading to drug efflux from the brain capillary endothelial cell to the lumen. Our study suggests that VA suppresses this seizure-mediated transcriptional activation of P-gp by downregulating COX-2, thus reducing its own efflux and enhancing delivery to the brain producing increased efficacy in the responders.

Based on our findings, we propose that during seizures, high levels of glutamate increase COX-2 activity by binding to endothelial NMDA receptor which results in increased production of PGE_2_ activating the EP1 receptor (Fig. 6). This triggers an unidentified cascade of regulatory proteins to signal transcriptional activation of P-gp, thereby causing enhanced efflux of the prescribed AEDs and hence poor efficacy. The anticonvulsant, VA subdues this P-gp upregulation by downregulating COX-2. In summary, VA may suppress seizure-mediated P-gp upregulation by downregulating COX-2 at the BBB leading to its increased delivery to the brain, therefore displaying enhanced efficacy in the responders. However, the same is not true for non-responders implying that the unidentified upstream mechanism by which VA is able to downregulate COX-2 is functional only in the responders and required further investigation of the upstream pathway by which VA imparts alteration in COX-2 expression. In addition, evidence regarding whether the described mechanism actually leads to increased brain delivery of VA is missing from our study. Therefore, for further in-depth understanding of the detailed underlying mechanism, validation of our findings in appropriate *in vivo* models is required to establish a causal relationship. Additionally, investigating the adjunctive effect of COX-2 inhibitors on the efficacy of prescribed AEDs in non-responders may reveal the potential adjunctive effect of COX-2 inhibitors in increasing AED efficacy.

Another gene which remained significantly differentially expressed after FDR adjustment was *GNLY* which encodes the cytotoxic molecule, granulysin. Granulysin is an anti-microbial and anti-tumorigenic molecule which is expressed in response to viral, bacterial, fungal, and parasitic infections and against tumor cells^67^. In regard to AED treatment, the mRNA as well as protein levels of this gene were earlier reported to be highly increased in blister cells from skin lesions of patients with AED-induced Stevens-Johnson syndrome (SJS) and toxic epidermal necrolysis (TEN) in comparison to that expressed in the peripheral blood mononuclear cells (PBMCs) of healthy controls^68^. The same study observed no difference in *GNLY* mRNA expression in the PBMCs of patients and healthy controls. In contrast, our study observed an upregulation in *GNLY* mRNA expression in the blood samples of “VA Non-responders”. Besides, none of the patient in this group showed any symptom of SJS/TEN even after a median treatment duration of 20.5 months (Table 1) indicating blister cell-specific association of *GNLY* upregulation in SJS/TEN cases. For further investigation of the role of *GNLY*, pharmacogenomic studies involving patients with AED-induced SJS/TEN need to be performed.

Our study has some limitations. First, the study is limited by a small sample size. A sample size calculation based on the expression values of all the DEGs using the SSPA package in BioConductor^69^ indicated the requirement of at least 17 samples in each group to achieve a statistical power of 80%. However, the study is a preliminary investigation where a small sample size might deliver findings with a greater scientific value relative to the expense of such high-throughput techniques^70^. Future gene expression studies will require validation in larger sample sizes to establish the robustness of the current findings. Second, our work examined peripheral blood gene expression profiles for a central neurological disease, epilepsy. Previous gene expression studies have reported several biological pathways to be shared between the blood and the brain^71,72^. To relate such similarities to pathological conditions, a comparative analysis of gene expression profiles between blood and brain in different neurological diseases needs to be performed in future. Third, the *in vitro* work was performed in hCMEC/D3 cell line which, over the years, has developed an infamously poor reputation in reflecting the BBB properties due to lack of tight junction formation by these cells leading to high permeability and low transendothelial electrical resistance (TEER) values, and low P-gp expression in comparison to mouse brain endothelial cells making these cells unsuitable for drug/transporter studies^73,74^. However, the cell line is, so far, the only available human brain endothelial cell line and we found the P-gp expression in these cells adequate enough to perform gene regulation studies. Lastly, our study employed a microarray-based platform to identify changes in gene expression. Though microarray has similar performance to RNA-Seq in clinical endpoint prediction showing a highly correlation in the generated data^75^, the latter surpasses it in detecting low abundance RNA transcripts, detecting DEGs with very high FC, distinguishing between biologically relevant isoforms, and identifying genetic variants^76^, and thus, may generate more insight into the clinically important biological mechanisms. The strength of the current study is the stringent selection criteria for enrolling patients and for selecting the DEGs after the appropriate adjustment of the confounding variables.

In conclusion, our study provides clinical evidence on the association between COX-2 expression profiles and VA efficacy in PWE. Our *in vitro* work indicated this association to be an effect of VA treatment demonstrating COX-2-dependent P-gp downregulation by VA. A pan inhibition of P-gp may lead to deleterious effects^26^. Similarly, adopting a polypharmaceutical approach to target the multiple EP receptors may produce undesired effects due to drug-drug interactions^77^. Targeting an upstream molecule such as, COX-2 may demonstrate a potential future therapeutic strategy for epilepsy management^78^. Therefore, COX-2 can be considered a potential neurotherapeutic target for developing a novel pharmacological antiepileptic treatment. However, to gather evidence on its effect on the brain delivery of prescribed AEDs, *in vivo* validation of our findings and exploration of the underlying molecular mechanism connecting COX-2 to P-gp may provide in-depth understanding to establish a causal relationship. Future clinical studies should investigate the effect of COX-2 inhibitors in adjunction to prescribed AEDs in large-sample, randomized, controlled trials with a parallel assessment of plasma PGE_2_ levels to substantiate the potential adjunctive application of COX-2 inhibitors for epilepsy treatment. Our work delivers preliminary data that may help in overcoming pharmacoresistance and developing an improved and better efficacious treatment for epilepsy.

## Methods

### Patient enrolment

Our subjects were PWE of North Indian ethnicity, aged 10–40 years, who were enrolled and followed-up at the Outpatient Department of Neurology, Institute of Human Behaviour and Allied Sciences (IHBAS), Delhi, India after obtaining informed consents. Patients with gross neurological deficits such as mental retardation and/or motor deficits; with imaging abnormalities including the presence of a tumor, tuberculoma, multiple neurocysticercosis, vascular malformations, and atrophic lesions; with severe hepatic/renal disorders or other medical illnesses; on medications other than AEDs; and pregnant women with epilepsy were excluded^3^. The study protocol was approved by the institutional ethics committee of Indian Council of Medical Research, IHBAS, and Institute of Genomics and Integrative Biology. All research was performed in accordance with relevant guidelines and regulations. Diagnosis and treatment were performed by experienced neurologists. Since the patients for the previous studies^3,50^ as well as the current study from our group were recruited from 2005–2015, the old ILAE scheme for seizure and epilepsy classification^79,80^ was followed. The patients in the current study fall into the idiopathic epilepsy group according to the old classification^80^ which corresponds to the epilepsies of genetic etiology in the 2017 classification system^81^, as proposed by Berg *et al*.^82^. Seizure episodes in the patients and surrounding history were reported by an eye-witness or a family member or a friend. A total of 23 patients with idiopathic/genetic epilepsy receiving VA monotherapy were selected for the current study and were categorized into “VA Responders” if remained seizure-free during the one-year follow-up period and “VA Non-responders” if experienced at least three seizures during the same period. At follow-up completion, 7–9 ml of blood sample was withdrawn from each patient with a single venipuncture in a VACUETTE® EDTA Tube (Greiner Bio-One, USA). Besides, blood samples were also obtained from 13 newly diagnosed “Drug-free” patients with idiopathic epilepsy (control group)^50^.

### RNA processing and microarray hybridization

Total RNA was isolated from the blood samples on the same day of collection using the TRIzol reagent (Invitrogen, CA, USA). The isolated RNA samples were then purified and quantified as described previously^50^. All of the samples achieved an A_260_/A_280_absorbance ratio >1.9 and a 28 S/18 S rRNA ratio >1.8. Biotin-labeled complementary RNA (cRNA) was produced by amplifying the purified RNA using the Illumina TotalPrep RNA Amplification Kit (Ambion, USA), quantified spectrophotometrically, and later hybridized to the Illumina HT-12 Expression Beadchip v4 consisting 47,231 probes as per the manufacturer’s instructions.

### *In silico* analysis

As discussed previously^50^, expression data of all the probes for each sample were extracted using GenomeStudio 2011. *In silico* analysis was performed in R statistical computing. Firstly, background subtraction and filtration of probes were performed. The expression values of the expressed probes were then log_2_ transformed, followed by normalization using SNM method where the effect of any confounding variable other than the biological variable in observation i.e. drug response, was adjusted. Finally, the linear empirical Bayes model in the Bioconductor “limma” package was applied to perform differential expression analysis where genes with FC > 2 and p < 0.05 were considered as significant. The multiplicity of tests were accounted by adjusting the p-values with Benjamini and Hochberg’s FDR < 0.1. In addition, ROC analysis was performed to evaluate the diagnostic performance of identified DEG using the pROC package for R.

### qRT-PCR validation

All of the identified DEGs were technically validated with the same set of samples in qRT-PCR using the LightCycler® 480 Instrument (Roche, Switzerland). All the primers were designed using Primer3 Input (v.0.4.0) (Supplementary Table 2). The RNA samples were converted to cDNA using the RevertAid H Minus First Strand cDNA Synthesis Kit (Thermo Fisher Scientific, USA). The resultant cDNA samples were then subjected to qRT-PCR with SYBR Green chemistry to assess the mRNA levels of the DEGs. The FC for each gene was calculated as described previously^50^. For the statistical analysis, unpaired *t*-test was used considering p < 0.05 as significant.

### Plasma prostaglandin E2 (PGE2) measurement

An ELISA assay was performed to measure plasma PGE2 levels of the patients using DetectX Prostaglandin E_2_ Enzyme Immunoassay kit (Arbor Assays, MI, USA) according to the manufacturer’s directions. Kruskal-Wallis with post-hoc Dunn test adjusted by Benjamini-Hochberg FDR method was used to compare the PGE2 levels between test groups and control group. Data was represented in median. p < 0.05 was considered statistically significant.

### Cell culture and treatments

hCMEC/D3 cells (P25) were procured from CEDARLANE Laboratories Corporation, Burlington, Canada. Cells were cultured in collagen type I (75 μg/ml) (Invitrogen, CA, USA) coated flasks, maintained in Endothelial Basal Medium-2 (EBM-2) (Lonza, Switzerland) supplemented with 5% foetal bovine serum (FBS) (Invitrogen, CA, USA), 1% penicillin-streptomycin (Invitrogen, CA, USA), 1/100 chemically defined lipid concentrate (Invitrogen, CA, USA), 10 mM HEPES (Sigma-Aldrich, St. Louis, MO), 1.4 μM hydrocortisone (Sigma-Aldrich, St. Louis, MO), 5 μg/ml ascorbic acid (Sigma-Aldrich, St. Louis, MO), and 1 ng/ml basic fibroblast growth factor (bFGF) (Invitrogen, CA, USA), and housed in an incubator at 37 °C, 5% CO2 according to the distributor’s recommended protocol. The cells were passaged till P35. Glutamate, ActD, CCX, CHX, rhodamine 123, VA, and VPL were purchased from Sigma-Aldrich, St. Louis, MO. NMDA receptor antagonist, MK-801 and EP1 receptor antagonist, SC-51089 were purchased from Abcam, Cambridge, MA. Glutamate and MK-801 were dissolved in water, ActD in ethanol and the rest of the chemicals were dissolved in dimethyl sulfoxide (DMSO). Vehicle control (VC) contained either water or DMSO or their combination as per the solvent in which the chemicals were dissolved.

### Cell viability test

Cell viability upon different treatments was measured using the colorimetric MTT (AMRESCO, USA) assay. Cells were seeded at 2000 cells/well in 96-well plate and incubated at 37 °C overnight followed by treatment with different chemicals under study for 24 h. 5 mg/mL MTT was added and the assay was performed as described earlier^83^.

### RNA isolation and qRT-PCR in hCMEC/D3 cells

Similar protocols were followed for RNA isolation and qRT-PCR analysis for hCMEC/D3 cells as that for the blood samples. Primers for COX-2, EP1, and P-gp mRNA were designed using Primer3 Input (v.0.4.0) (Supplementary Table 2).

### Immunoblotting

Cells were treated with chemicals under study followed by protein extraction using lysis buffer (50 mM Tris–HCl, pH 8, 150 mM NaCl, 1% NP-40, 0.5% sodium deoxycholate, 0.1% SDS) which is quantified by Pierce BCA Protein Assay Kit (Thermo Fischer Scientific, USA). Deglycosylation was performed prior to electrophoresis using PNGase F (New England Biolabs) for heavily glycosylated proteins. Equal amounts of protein extracts were electrophoresed on 8% and 10% sodium dodecylsulfate-polyacrylamide gel electrophoresis (SDS-PAGE) gel followed by transfer to nitrocellulose membrane. The membranes were incubated overnight at 4 °C with anti-COX-2 (Abcam, Cambridge, MA), anti-P-gp (Cell signaling technology, MA, USA), anti-EP1 (Abcam, Cambridge, MA), anti-Vinculin (Santa Cruz Biotechnology, CA, USA) and anti-HSC70 (Santa Cruz Biotechnology, CA, USA) antibodies. After incubation with horseradish peroxidase-conjugated secondary antibodies (GeNei, Bangalore, India; Santa Cruz Biotechnology, CA, USA), the bands were visualized using ECL detection reagents (Pierce) on Chemi Doc™ MP Imaging system (Bio-Rad, Hemel Hempstead, UK) and quantified with the AlphaImager 3400 (Alpha InnoTech corporation, Sam Leandro, CA) software. The intensities of the test genes under study were normalized using either vinculin or HSC70.

### COX-2 functional assay

After treating the cells with different chemicals, total protein was extracted from the cells as described above. COX-2 activity was assayed using COX Activity Assay Kit (Cayman Chemical, MI, USA) according to the manufacturer’s protocol.

### Rhodamine 123 efflux assay

After treatment with different chemicals, cells were washed with 1X Phosphate Buffered Saline (PBS). Opti-MEM medium having P-gp substrate, 10 µM rhodamine 123, was added for 1 hour at 37 °C in 5% CO_2_ to allow its uptake. Media was discarded and cells were washed with ice-cold PBS thrice, followed by incubation for another 1 hour at 37 °C in 5% CO_2_ in Opti-MEM medium, to allow efflux to occur. Efflux was stopped by washing with ice-cold PBS thrice and cells were lysed with 1% Triton X-100 at 37 °C for 15 min. Fluorescence intensities were measured using Infinite® 200 PRO multimode plate reader (Tecan, Switzerland) with excitation and emission wavelengths of 485 and 538 nm, respectively. Functional activity was measured as absolute fluorescence intensity in cell lysate per mg of total protein.

### ATPase assay

To assess the substrate relationship of VA with P-gp, MDR1/P-gp PREDEASY ATPase kit (Solvo Biotechnology, Budaors, Hungary) was used as per the protocol described by the manufacturer. The assay measures ATPase activity in isolated insect Sf9 cell membranes overexpressing human P-gp in the presence of VA, therefore quantifying P-gp-specific ATPase activity. In addition, the inhibition assay part of this experiment examines the inhibitory potential of a test drug on the P-gp ATPase activity induced by a known substrate. The results were expressed as vanadate-sensitive ATPase activities.

### Statistical analysis of hCMEC/D3 data

Data were represented as mean ± standard deviation. To determine statistical significance, either student’s *t*-test or one-way ANOVA with Tukey honest significant difference (HSD) test was performed as appropriate. p < 0.05 was considered statistically significant.

## Supplementary information

Supplementary Information.

## Data Availability

The microarray data is available at the Gene Expression Omnibus (GEO) repository under accession number GSE143272.
