# Supplementary material for: Downregulation of peripheral PTGS2/COX-2 in response to valproate treatment in patients with epilepsy
Source: Sci Rep. 2020 Feb 13;10:2546. doi: 10.1038/s41598-020-59259-x (PMC7018850; doi:10.1038/s41598-020-59259-x)
Supplement: Supplementary file 1 — Supplementary Information. [file 41598_2020_59259_MOESM1_ESM.pdf]

**Title: Downregulation of peripheral *PTGS2*/COX-2 in response to valproate treatment in patients with epilepsy**

**Running title:** *PTGS2* downregulation in response to valproate

**Authors:** Chitra Rawat[1,2], Rintu Kutum[1,2], Samiksha Kukal[1,2], Ankit Srivastava[1,3], Ujjwal Ranjan Dahiya[1,2], Suman Kushwaha[4], Sangeeta Sharma[4], Debasis Dash[1,2], Luciano Saso[5], Achal K Srivastava[6], Ritushree Kukreti[1,2,*]

[1]Institute of Genomics and Integrative Biology (IGIB), Council of Scientific and Industrial Research (CSIR), Mall Road, Delhi-110007, India

[2]Academy of Scientific and Innovative Research (AcSIR), Council of Scientific and Industrial Research (CSIR), Mall Road, Delhi-110007, India

[3]Department of Pharmacology, Faculty of Pharmacy, Jamia Hamdard, Delhi-110062, India

[4]Institute of Human Behavior & Allied Sciences (IHBAS), Dilshad Garden, Delhi-110095, India

[5]Department of Physiology and Pharmacology "Vittorio Erspamer", Sapienza University, Rome, Italy

[6]Department of Neurology, All India Institute of Medical Sciences, Ansari Nagar, Delhi-110029, India

*Correspondence to Ritushree Kukreti, PhD, Genomics and Molecular Medicine Unit, Institute of Genomics and Integrative Biology (IGIB), Council of Scientific and Industrial Research (CSIR), Mall Road, Delhi-110007, India. Tel: + 91 11 27662202; fax: + 91 11 27667471; e-mail: ritus@igib.in

**Supplementary Table 1: Common DEGs between “VA Responders” and “VA Non-responders” (FC>2, $P_{uncorrected}$<0.05)**

| Probeset ID | Gene Symbol | Gene name | Cytoband | VA Responders | | | VA Non-responders | | | Direction |
|---|---|---|---|---|---|---|---|---|---|---|
| | | | | P Value | FDR | Fold Change | P Value | FDR | Fold Change | |
| ILMN_1779071 | *FEZ1* | Fasciculation and elongation protein zeta 1 (zygin I) | 11q24.2 | <0.0001 | 0.0154 | 2.09 | <0.0001 | 0.0081 | 2.43 | UP |
| ILMN_1791253 | *FBXL13* | F-box and leucine-rich repeat protein 13 | 7q22.1 | <0.0001 | 0.0236 | -2.21 | 0.0009 | 0.0920 | -2.33 | DOWN |
| ILMN_1780546 | *OSM* | Oncostatin M | 22q12.2 | <0.0001 | 0.0236 | -2.48 | 0.0016 | 0.0998 | -2.12 | DOWN |
| ILMN_1806056 | *CEACAM8* | Carcinoembryonic antigen-related cell adhesion molecule 8 | 19q13.2 | 0.0286 | 0.1919 | 2.30 | 0.0330 | 0.1953 | 2.34 | UP |

**Supplementary Fig 1: ROC curves of blood *PTGS2*/COX-2 mRNA expression in the epilepsy cohort**

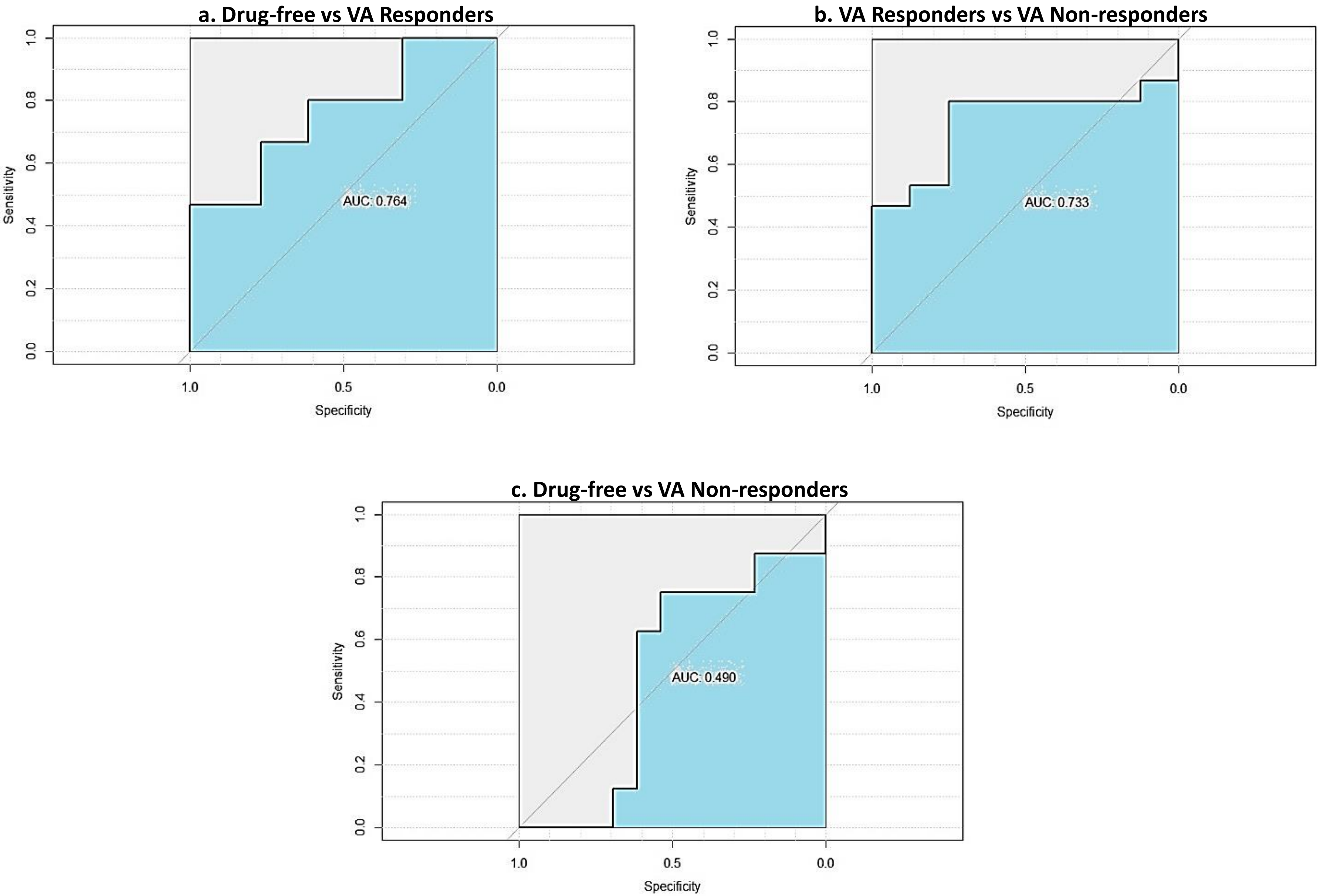

## Supplementary Table 2: List of primer sequences specific to selected DEGs

| Gene | Forward Primer | Reverse Primer |
|---|---|---|
| *PTGS2* (COX-2) | 5'-CCTGTGCCTGATGATTGC-3' | 5'-CTGATGCGTGAAGTGCTG-3' |
| *MPO* | 5'- CCACCAAAACCGATCACCAT-3' | 5'-CACTCCTCGCCTGCATCAT-3' |
| *DEFA3/DEFA1B/DEFA1/LOC653600* | 5'-TCCTTGCTGCCATTCTCCTG-3' | 5'-TGAGCCTGGATGCTTTGGAG-3' |
| *CTSG* | 5'-CCTGTGCCTGATGATTGC-3' | 5'-CTGATGCGTGAAGTGCTG-3' |
| *CEACAM6* | 5'-ACCGTCCAGGGGAAAATCTG-3' | 5'-CAGGAGCACTTCCAGAGACTG-3' |
| *ELANE* | 5'-CCACCCGGCAGGTGTTC-3' | 5'-GTGGCCGACCCGTTGAG-3' |
| *DEFA4* | 5'-GTCTGCTCTTGCAGATTAGTATTCTG-3' | 5'-TTAATCGACACGCGTGCAGCAGTAT-3' |
| *GNLY* | 5'-AGGCTCCCTGCCCATAAAAC-3' | 5'-TCAGGGCTCAGACGAGAGAA-3' |
| *VAV3* | 5'-CATCCATTGCAAGGTGCTGC-3' | 5'-AGAAACTGGGACATCTGCGG-3' |
| *LY6E* | 5'-AGGACAGGCTGCTTTGGTTT-3' | 5'-CACGCAGTAGTTGTCCTGGT-3' |
| *SNHG8* | 5'-AAGTTTACAAGCATGCGCGG-3' | 5'-TCAAACTGACGGTTCTCGGG-3' |
| *ORM1* | 5'-CAGTCCTGAGCCTCCTACCT-3' | 5'-GTCCTCTGTCTTGTTGGGGG-3' |
| *PGM5* | 5'-CAGGCTGCAGATTCCCTCC-3' | 5'-TCAGTCGTCCAATCCCGTTG-3' |
| *PTGER1* (EP1) | 5'-GTGTACATCCTACTGCGCCA-3' | 5'-CTTAGTCGTTGGGCCTCTGG-3' |
| *ABCB1* (P-gp) | 5'-GCCTGGCAGCTGGAAGACAAATAC-3' | 5'-ATGGCCAAAATCACAAGGGTTAGC-3' |
| *18S rRNA* | 5'-AACCCGTTGAACCCCATT-3' | 5'-CCATCCAATCGGTAGTAGCG-3' |
